# Positive Association between the Triglyceride-Glucose Index and Hyperuricemia in Chinese Adults with Hypertension: An Insight from the China H-Type Hypertension Registry Study

**DOI:** 10.1155/2022/4272715

**Published:** 2022-02-12

**Authors:** Chao Yu, Tao Wang, Wei Zhou, Lingjuan Zhu, Xiao Huang, Huihui Bao, Xiaoshu Cheng

**Affiliations:** ^1^Center for Prevention and Treatment of Cardiovascular Diseases, the Second Affiliated Hospital of Nanchang University, Nanchang, Jiangxi, China; ^2^Jiangxi Provincial Cardiovascular Disease Clinical Medical Research Center, Nanchang, Jiangxi, China; ^3^Jiangxi Sub-Center of National Clinical Research for Cardiovascular Diseases, Nanchang, Jiangxi, China; ^4^Department of Cardiovascular Medicine, the Second Affiliated Hospital of Nanchang University, Nanchang, Jiangxi, China

## Abstract

**Background:**

Previous studies have revealed the triglyceride-glucose (TyG) index is closely related to hyperuricemia in the general population. However, this relationship in hypertensive patients has not been reported. The aims of this study are to investigate the relationship of the TyG index and hyperuricemia in adult Chinese hypertension.

**Methods:**

The China H-type Hypertension Registration (an observational, noninterventional, and real-world study) was conducted from March 01, 2018, to August 31, 2018, in 16 communities in Wuyuan of China. The TyG index was calculated from fasting triglycerides (mg/dL) and fasting glucose (mg/dL)/2. Hyperuricemia was categorized by uric acid concentration ≥420 *μ*mol/L (7 mg/dL).

**Results:**

Overall average TyG index of 13,060 adults with hypertension was 8.87; age was 63.81 years. The TyG index was positively correlated with serum uric acid by multiple linear regression analyses (*β* = 38.03; 95% CI: 34.55 to 41.51). Coincidentally, logistic analyses also demonstrated the TyG index had a positive correlation with hyperuricemia (OR = 2.04; 95% CI: 1.87 to 2.24). Strong linear associations of the TyG index with serum uric acid and hyperuricemia were confirmed by restricted cubic spline analysis. Compared with subjects in the lowest quartile (7.13 to 8.44) of the TyG index, subjects in the 2^nd^–4^th^ quartiles had 1.25 (95% CI: 1.10 to 1.41), 1.63 (95% CI: 1.43 to 1.86), and 2.79 (95% CI: 2.41 to 3.24) times greater incident risk of developing hyperuricemia. The TyG index had significant correlations between male patients and hyperuricemia (OR = 2.01) by subgroup analysis.

**Conclusion:**

Positive associations were found between the TyG index and serum uric acid and between the TyG index and hyperuricemia in adults with hypertension. This trial was registered at clinicaltrials.gov as ChiCTR1800017274.

## 1. Introduction

The worldwide prevalence of hyperuricemia has been rapidly increasing over the last decades [1, 2]. Hyperuricemia can cause the saturation of the weakly water-soluble urate salt and precipitate out in the form of monosodium urate crystals, causing kidney stones and gout. In addition, hyperuricemia is also closely related to many diseases including hypertension [3], major cardiovascular and coronary events [4], type 2 diabetes [5], chronic kidney disease [6], obesity [7], and metabolic syndrome [8].

The triglyceride-glucose (TyG) index was first reported in 2008 [9] and compared with the HOMA-IR index, indicating that the TyG index can be used as a reliable, inexpensive, and simple substitute for insulin resistance [10, 11]. Insulin resistance is defined as a profound dysregulation of the insulin signaling system and represents a state of the impaired ability of peripheral tissues to respond to the physiological levels of insulin.

Recent evidence has emerged from several large epidemiological studies that hyperuricemia is related to insulin resistance [12–14]. Although previous studies have investigated the association between TyG and hyperuricemia [15–17] among general populations, to our knowledge, there are currently no published studies focusing on hypertensive populations. Additionally, epidemiological studies have shown that the prevalence of hyperuricemia is higher in hypertensive patients [18, 19]. Hyperuricemia and IR are important risk factors for essential hypertension [20, 21]. Considering that this high-risk group has a greater risk of cardiovascular disease, it is necessary to clarify the precise relationship between the TyG index and hyperuricemia in hypertensive patients. Accordingly, this study was carried out to examine the relationship between the TyG index and the risk of hyperuricemia and to provide a basis for identifying the high-risk population of hyperuricemia.

## 2. Methods

### 2.1. Participants

Data analyzed in this study were registered previously (the China H-type Hypertension Registry Study, registration no. ChiCTR1800017274). Previous studies described the exclusion criteria and the method of data collection [22, 23]. This was a real-world, noninterventional, cross-sectional study conducted in March 2018 in Wuyuan of China [22, 23]. A total of 14,234 adults (aged 18 years or over) with hypertension were screened through a census. Hypertension was defined as systolic blood pressure (SBP) ≥140 mmHg or/and diastolic blood pressure (DBP) ≥90 mmHg when blood pressure was measured in a resting and sitting position or taking antihypertensive medications or self-report of a hypertensive diagnosis. The three consecutive measurements were then averaged to obtain the final blood pressure values. The exclusion criteria were described as follows: (1) mental or neurological abnormalities that prevent collaboration with the investigation; (2) unable to complete follow-up due to poor adherence or planned to relocate recently; (3) the participants assessed by the study physicians as unsuitable for inclusion or long-term follow-up; and (4) participants with lipid-lowering drugs were excluded, when the drugs' impact on biochemical indicators was considered [22, 23].

**Figure 1:**
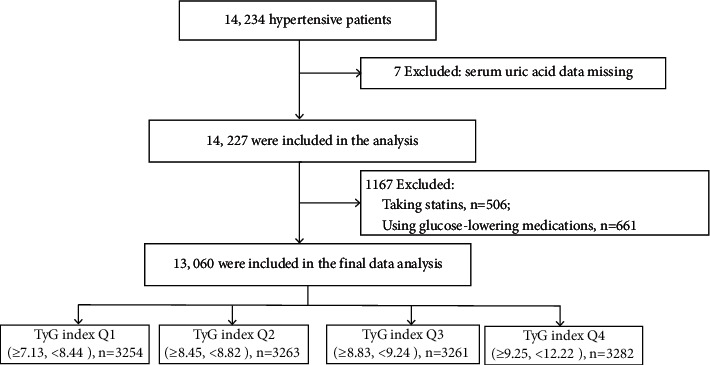
Flowchart of the study population inclusion.

Finally, a total of 13,060 subjects were included in this study. The flowchart in Figure 1 was used to show detailed information about the sample size of the subjects and the exclusion criteria in the current research. All participants have signed an informed consent form. Ethics approval was given by the Biomedical Ethics Committee of Anhui Medical University [22, 23].

### 2.2. Data Collection

Data were collected by our team consisting of clinical cardiologists, local public health physicians, nurses, and volunteers. Face-to-face interviews and questionnaires were used to investigate, including physical measurements, laboratory examinations, and auxiliary examinations. The content of the questionnaire includes basic personal information (age, gender, ethnicity, occupation, education, marital status, etc.); living habits (smoking, drinking, diet, labor intensity, sleep duration, etc.); physical measurements including height, weight, waist circumference, and blood pressure; laboratory tests including blood homocysteine, blood lipids, blood sugar, bilirubin, blood routine, and urine routine; and auxiliary examinations including all patients with the electrocardiogram (ECG8322).

### 2.3. Laboratory Assays

Of all participants, we collected fasting venous blood samples after overnight fasting at the baseline; all samples were processed at the National Clinical Research Center Clinical Laboratory for Kidney Disease, Guangzhou, China [22, 23]. Various biomarkers (serum homocysteine, fasting glucose, fasting plasma lipids (total cholesterol, triglycerides, HDL-C, and LDL-C), and creatinine) were measured using automatic clinical analyzers (reagents from Guangdong Zhongshan Baijia Biotechnology Co., Ltd., using Beckman Coulter AU680) [22, 23]. Serum uric acid was measured by the uricase-peroxidase method; fasting blood glucose was assessed by the hexokinase method; cholesterol and serum triglycerides (TG) were measured by the oxidase method; serum HDL cholesterol and serum LDL cholesterol were analyzed by the direct method; serum homocysteine was determined enzymatically. We calculated the TyG index according to the following formula: TyG = ln [TG (mg/dL) × fasting glucose (mg/dL)/2] [22]. Chronic Kidney Disease Epidemiology Collaboration (CKD-EPI) equation was used to estimate glomerular filtration rate (eGFR) in adults [22, 24].

### 2.4. Definition of Hyperuricemia

Hyperuricemia diagnosis was made at serum uric acid ≥420 *μ*mol/L for all males and females, according to the latest guideline for the diagnosis and management of hyperuricemia and gout in China [25].

### 2.5. Statistical Analysis

Data of continuous variables and categorical variables were presented as the mean ± standard deviation/median (minimum, maximum) and percentage/numbers, respectively. The comparison of the baseline characteristics among different groups by TyG index quartiles (quartile 1, 7.1–8.5; quartile 2, 8.5–8.8; quartile 3, 8.8–9.3; and quartile 4, 9.3–12.2) was evaluated by chi-square (*x*^*2*^) tests (compared continuous variables) or analysis of variance (ANOVA) tests (categorical variables). The multivariate linear regression models (represent results by *β* and 95% confidence interval (CI)) and nonconditional logistic regression models (represent results by odds ratio (OR) and 95% CI) for major covariables adjusted for the main covariates in three models were designed to assess the independent association of the TyG index with serum uric acid and hyperuricemia. The covariates were selected on the basis of their clinical importance, statistical significance in the univariable analyses, and the estimated varables change of at least 10% of potential confounding effects. The penalized spline regression method (a fitted smoothing curve) and the generalized additive model (GAM) conducted the dose-response relationship for the TyG index with serum uric acid and hyperuricemia. In addition, we conducted subgroup analyses to explore the potential factors modifying the association.

All analyses used the statistical package R (http://www.r-project.org, version 4.1.2) and Empower 2.0 (R) (http://www.empowerstats.com). Two-tailed *P* < 0.05 was considered to be statistically significant.

## 3. Results

### 3.1. Baseline Characteristics

The quartile of the TyG index presented participants' characteristics in Table 1. A total of 13,060 subjects grouped according to the ranges of TyG index in quartile were Quartile 1 (7.1–8.5), Quartile 2 (8.5–8.8), Quartile 3 (8.8–9.3), and Quartile 4 (9.3–12.2), respectively. The mean (SD) age of the participants was 63.81 (9.47) years; 6223 were men (47.65%). The overall average TyG index was 8.87 ± 0.61. Average serum uric acid was 419.16 ± 120.69 *μ*mol/L. Age, female, HDL-C, and serum homocysteine tended to decrease with the TyG index, while BMI, waist circumference, SBP, DBP, fasting plasma glucose, total cholesterol, triglyceride, LDL-C, and eGFR tended to increase with the TyG index (*P* values < 0.05).

### 3.2. Relationship of the TyG Index among SUA and Hyperuricemia

**Figure 2:**
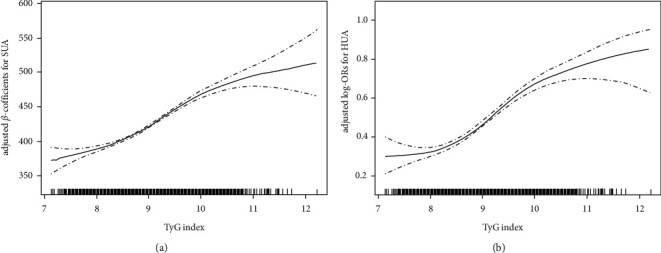
Dose-response association between the TyG index and SUA. (a) TyG index and SUA; (b) TyG index and hyperuricemia. All adjusted for age, gender, education, smoking, drinking, physical activity, waist circumference, BMI, SBP, DBP, eGFR, serum homocysteine, HDL-C, LDL-C, diabetes, antiplatelet drugs, and antihypertensive drugs.

Tables 2 and 3 separately display the effect of the TyG index quartile on the association between SUA and hyperuricemia. The increment of SUA is 38.03 *μ*mol/L (95% CI: 34.55, 41.51) with the TyG index per one unit increment, and the hyperuricemia risk odds ratio (OR) is 2.04 (95% CI: 1.87, 2.24) according to the estimation from multivariate linear regression models' indication. Moreover, the TyG index showed a significant positive relationship between SUA and the risk of elevated hyperuricemia (Figures 2(a) and 2(b)).

The multivariate linear regression models (models 1–3) adjusted for potential confounders show a positive association between the TyG index and SUA as demonstrated in Table 2. After the TyG index was assessed as quartiles, compared with subjects in the 1^st^ quartile, the, respectively, adjusted *β*-values of SUA for subjects in the 2^nd^–4^th^ quartiles were 12.36 (95% CI: 7.60, 17.1), 26.87 (95% CI: 21.71, 32.30), and 55.37 (95% CI: 49.59, 61.15) in the fully adjusted model (model 3). Uniformly, subjects of the TyG index in the 2^nd^–4^th^ quartiles had 1.25 (95% CI: 1.10 to 1.41), 1.63 (95% CI: 1.43 to 1.86), and 2.79 (95% CI: 2.41 to 3.24) times greater incident risk of developing hyperuricemia (*P* for trend < 0.001), compared with subjects in the lowest quartile (7.13 to 8.44), as presented in Table 3.

### 3.3. Subgroup Analyses

**Figure 3:**
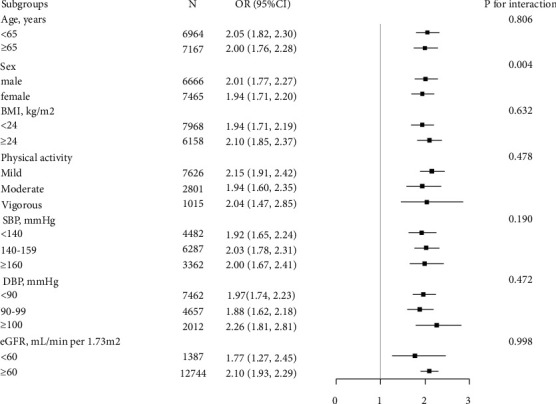
Subgroup analysis of the association between the TyG index and hyperuricemia. Adjusted for age, gender, education, smoking, drinking, BMI, waist circumference, physical activity, diabetes, eGFR, HDL-C, LDL-C, serum homocysteine, antiplatelet drugs, antihypertensive drugs, SBP, and DBP. The BP control maintained was defined as SBP <140 mmHg and DBP <90 mmHg.

Stratified analyses were conducted to reveal the association between the TyG index (per 1 unit increment) and hyperuricemia in different subgroups (Figure 3). No significant interactions were found in the following subgroups, including age (<65 vs. ≥65 years), physical activity (mild vs. moderate vs. vigorous), BMI (<24 vs. ≥24 kg/m^2^), eGFR (<60 vs. ≥60 mL/min per 1.73 m^2^), SBP (<140 vs. 140–159 vs. ≥160 mmHg), and DBP (<90 vs. 90–99 vs. ≥100 mmHg) subgroups (*P* values >0.05 for all), whereas the TyG index had significant correlations between male patients and hyperuricemia (males: OR = 2.01, 95% CI: 1.77–2.27; females: OR = 1.94, 95% CI: 1.71–2.20; *P* for interaction = 0.004).

## 4. Discussion

In this large population-based study, for the first time, we investigated the association between the TyG index and hyperuricemia in the hypertension population. After adjusting the confounding factors, we showed that the TyG index was independently related to SUA and hyperuricemia. The restricted cubic spline indicated that the association was linear in the whole range of TyG. Moreover, the findings suggested that the association seemed to be strong among those male participants and hypertension (*P* for interaction = 0.004).

In the epidemiological survey of the hypertensive population in this study, the prevalence rate of hyperuricemia in the hypertensive population was 44.8%, which was generally consistent with the results of previous studies [26].

Some recent studies have attempted to explore the association of the TyG index with SUA and hyperuricemia. Gu et al. [17] conducted a retrospective longitudinal analysis of 42387 people who had a physical examination and had no HUA at baseline. It was found that the HRs of men and women in the highest third quartile of TyG were 1.440 (95% CI: 1.254–1.654, *P* < 0.001) and 1.753 (95% CI: 1.314–2.337, *P* < 0.001), respectively. The study indicated that TyG had the potential to help with risk stratification and prevention of hyperuricemia, especially for different-sex patients. Similarly, Shi et al. [15] conducted a cross-sectional study involving 6466 general population. The results showed that when TyG was divided into quartiles, the risk of hyperuricemia in the highest quartile was 2.730 times higher than that in the lowest quartile, and it revealed a strong linear correlation between TyG and hyperuricemia. Liu et al. [16] analyzed and compared the relationship between three simple insulin resistance indexes (including TyG) and hyperuricemia. Regardless of the classification of BMI, TyG was closely related to hyperuricemia. Multiple regression analysis showed that TyG was significantly correlated with hyperuricemia (the OR of the highest quartile of women was 1.505 (95% CI: 1.311–1.727). The male was 1.646 (95% CI: 1.431–1.893). Similarly, Zhu et al. [27] compared the relationship between different simple insulin resistance indexes (including TyG) and hyperuricemia in 11098 subjects with normal BMI. The results showed that there was a significant positive correlation between TyG and uric acid levels (*P* < 0.001), and the OR of the highest quartile of TyG was 1.665 (95% CI: 1.147–2.418, *P* = 0.007) and 2.894 (95% CI: 1.486–5.637, *P*=0.002) for males and females, respectively. The results of our study are similar to the above results, which further show a stable positive association between the TyG index and hyperuricemia. Our research revealed larger regression coefficients and odds ratios of the TyG index associated with hyperuricemia in male patients compared with females. These results may be explained by the sex differences in fat distribution, glycolipid metabolism, and urate metabolism [28].

The mechanisms underlying the relationship between TyG and hyperuricemia are not yet fully understood. A possible explanation for it is that the TyG index is a combination index of blood lipids and fasting blood glucose. For example, William Gustavo et al. [29] assessed high uric acid levels to regulate oxidative stress, inflammation, and enzymes associated with glucose and lipid metabolism. de Oliveira and Burini [30] showed the process of synthesizing fatty acid (i.e., TG) that occurs in the liver is related to de novo synthesis of purines that accelerate the production of SUA; abnormal blood lipid metabolism can easily cause arteriosclerosis, including renal arteriosclerosis, reduce renal blood volume, lead to urate excretion disorders, and then increase uric acid levels. In addition, insulin resistance plays a causative role in the formation and development of hyperuricemia by inducing systemic inflammation, affecting lipid metabolism, causing kidney damage, and reducing renal uric acid excretion, and TyG is an indicator of insulin resistance. Studies also have shown that hyperuricemia can affect adipocytes by increasing monocyte chemoattractant protein and reducing the production of adiponectin, thereby contributing to insulin resistance and inflammation [31]. Thirdly, insulin resistance can cause secondary hyperinsulinemia, further stimulate the exchange of sodium and hydrogen ions in renal tubules, and increase the reabsorption of uric acid anions. It affects the excretion of Na in renal tubules and competitively inhibits the excretion of uric acid, increasing serum uric acid. Hence, Simental-Mendía et al. [32] found that elevated uric acid levels could be related to early disorders in the beta-cell function.

The strengths of our study include using standardized measurements for lifestyle factors, physical, laboratory tests, and relatively large population-based research. The present study still has limitations. First, the causality relationship between TyG and hyperuricemia in the hypertension population cannot be inferred from the cross-sectional design. Second, the questionnaire used for the study did not collect information on the use of low-uric acid drugs; our diagnosis of hyperuricemia may have been affected. However, considering that many previous studies have not reported the use of low-uric acid drugs in investigating hyperuricemia, we believe our results are still acceptable. Third, only Chinese adults with hypertension were included in our investigation. Hence, it would be restricted to extend to other people. Fourth, although our model has contained many covariates, residual mixing caused by unrecorded risk factors (socioeconomic information and diet structure) of hyperuricemia may cause bias to our findings.

## 5. Conclusion

In a nutshell, this study revealed a positive association between TyG and the risk of hyperuricemia in people with hypertension. In addition, the TyG index represents a simple, economically-friendly, and independent risk factor, which may act as a potential obtainable indicator in hypertension with hyperuricemia management.

## Figures and Tables

**Table 1:** Clinical characteristics of the study population according to the TyG index.

	Q1 (7.13–8.44)	Q2 (8.45–8.82)	Q3 (8.83–9.24)	Q4 (9.25–12.22)	*P* value
*N*	3254	3263	3261	3282	
Age (y)	66.42 ± 9.38	64.73 ± 9.38	63.03 ± 9.16	61.08 ± 9.10	<0.001
Male, *n* (%)	2167 (62.72)	1709 (47.43)	1423 (40.22)	1367 (38.67)	<0.001
Education, *n* (%)					<0.001
<High school	2456 (92.68)	2443 (93.07)	2387 (91.14)	2451 (90.04)	
≥High school	194 (7.32)	182 (6.93)	232 (8.86)	271 (9.96)	
Current smoking, *n* (%)	1157 (35.57%)	870 (26.66%)	688 (21.10%)	734 (22.37%)	<0.001
Current drinking, *n* (%)	948 (29.14%)	714 (21.89%)	615 (18.87%)	648 (19.75%)	<0.001
BMI (kg/m^2^)	21.81 ± 4.10	23.03 ± 3.54	24.07 ± 3.28	25.09 ± 3.22	<0.001
Waist circumference (cm)	78.55 ± 9.06	82.17 ± 9.49	85.24 ± 9.54	88.07 ± 8.63	<0.001
Physical activity, *n* (%)					0.688
Mild	1767 (66.68)	1759 (67.01)	1708 (65.22)	1824 (67.01)	
Moderate	652 (24.60)	631 (24.04)	684 (26.12)	658 (24.17)	
Vigorous	231 (8.72)	235 (8.95)	227 (8.67)	240 (8.82)	
SBP (mmHg)	147.48 ± 18.20	148.82 ± 17.66	148.43 ± 17.35	149.53 ± 18.10	<0.001
DBP (mmHg)	87.50 ± 11.01	88.67 ± 10.69	89.26 ± 10.47	91.23 ± 10.67	<0.001
Fasting glucose (mmol/L)	5.45 ± 0.60	5.77 ± 0.71	6.02 ± 0.91	6.88 ± 2.07	<0.001
Total cholesterol (mmol/L)	4.68 ± 0.94	5.10 ± 0.95	5.36 ± 1.06	5.58 ± 1.21	<0.001
Triglyceride (mmol/L)	0.82 ± 0.17	1.25 ± 0.18	1.77 ± 0.30	3.26 ± 1.57	<0.001
Serum homocysteine, *μ*mol/L	18.25 ± 11.19	18.58 ± 11.89	17.58 ± 10.77	17.63 ± 10.46	<0.001
HDL-C (mmol/L)	1.74 ± 0.46	1.64 ± 0.41	1.54 ± 0.38	1.40 ± 0.37	<0.001
LDL-C (mmol/L)	2.54 ± 0.65	2.92 ± 0.68	3.19 ± 0.76	3.33 ± 0.85	<0.001
eGFR (ml/min per 1.73 m^2^)	87.66 ± 20.06	88.08 ± 19.86	89.11 ± 19.45	89.07 ± 20.21	0.005
TyG index	8.15 ± 0.23	8.64 ± 0.11	9.02 ± 0.12	9.68 ± 0.39	<0.001
Serum uric acid, *μ*mol/L	398.34 ± 112.00	406.44 ± 116.60	419.19 ± 118.91	452.41 ± 127.58	<0.001
Hyperuricemia, *n* (%)	1225 (37.65%)	1324(40.58%)	1457 (44.68%)	1846 (56.25%)	<0.001
Diabetes, *n* (%)	86 (2.64%)	230 (7.05%)	399 (12.24%)	1061 (32.33%)	<0.001
Antihypertensive drugs, *n* (%)	1980 (60.87%)	2095 (64.20%)	2069 (63.47%)	2131 (64.95%)	0.004
Antiplatelet drugs, *n* (%)	65 (2.0%)	73 (2.24%)	65 (1.99%)	68 (2.07%)	0.890

Values are *N* (%), mean ± SD. BMI: body mass index (calculated as weight in kilograms divided by height in meters squared); SBP: systolic blood pressure; DBP: diastolic blood pressure; HDL-C: high-density lipoprotein cholesterol; LDL-C: low-density lipoprotein cholesterol; eGFR: estimated glomerular filtration rate; TyG: triglyceride-glucose.

**Table 2:** Association between the TyG index and SUA in different models.

TyG index	Serum uric acid, *μ*mol/L, *β* (95% CI)			
	Crude model	Model I	Model II	Model III
Per 1 unit increase	34.6 0 (31.25, 37.94)	37.9 0 (34.48, 41.32)	43.74 (40.44, 47.04)	38.03 (34.55, 41.51)
Quartiles				
Q1	0.00	0.00	0.00	0.00
Q2	8.10 (2.33, 13.88)	9.67 (3.90, 15.44)	19.67 (14.43, 24.90)	12.36 (7.60, 17.13)
Q3	20.85 (15.08, 26.63)	24.00 (18.20, 29.81)	37.26 (31.83, 42.69)	26.87 (21.71, 32.30)
Q4	54.07 (48.31, 59.84)	59.03 (53.16, 64.90)	68.85 (63.20, 74.49)	55.37 (49.59, 61.15)
*P* for trend	<0.001	<0.001	<0.001	<0.001

Model I: adjusted for age. Model II: adjusted for sex, age, BMI, SBP, DBP, education, physical activity, waist circumference, current drinking, and current smoking. Model III: adjusted for sex, age, BMI, SBP, DBP, education, physical activity, waist circumference, current drinking, current smoking, HDL-C, LDL-C, serum homocysteine, eGFR, diabetes, antiplatelet drugs, and antihypertensive drugs. TyG: triglyceride glucose; SUA: serum uric acid; CI: confidence interval.

**Table 3:** Association between the TyG index and HUA in different models.

TyG index	Hyperuricemia, OR (95% CI)			
	Crude model	Model I	Model II	Model III
Per 1 unit increase	1.62 (1.53, 1.72)	1.70 (1.60, 1.80)	2.07 (1.93, 2.22)	2.04 (1.87, 2.24)
Quartiles				
Q1	1.00	1.00	1.00	1.00
Q2	1.13 (1.02, 1.25)	1.12 (1.04, 1.28)	1.40 (1.25, 1.56)	1.25 (1.10, 1.41)
Q3	1.34 (1.21, 1.48)	1.39 (1.26, 1.54)	1.87 (1.66, 2.10)	1.63 (1.43, 1.86)
Q4	2.13 (1.93, 2.35)	2.27 (2.05, 2.52)	3.14 (2.78, 3.54)	2.79 (2.41, 3.24)
*P* for trend	<0.001	<0.001	<0.001	<0.001

Model I: adjusted for age. Model II: adjusted for sex, age, BMI, SBP, DBP, education, physical activity, waist circumference, current drinking, and current smoking. Model III: adjusted for sex, age, BMI, SBP, DBP, education, physical activity, waist circumference, current drinking, current smoking, HDL-C, LDL-C, serum homocysteine, eGFR, diabetes, antiplatelet drugs, and antihypertensive drugs. TyG: triglyceride glucose; HUA: hyperuricemia; CI: confidence interval.

## Data Availability

Previously reported data were used to support this study. These prior studies (and datasets) are cited at relevant places within the text as references [19, 20].
